# Effect of fecal microbiota transplantation on gut microbiota functional profile in recipients of allogeneic hematopoietic cell transplantation

**DOI:** 10.1080/19490976.2025.2551882

**Published:** 2025-08-27

**Authors:** Maryam Ebadi, Swetha Reddi, Liliia Senyshyn, Samuel S. Minot, Ted Gooley, Amanda J. Kabage, Stephanie J. Lee, Geoffrey R. Hill, Alexander Khoruts, Armin Rashidi

**Affiliations:** a Department of Radiation Oncology, University of Washington, Seattle, WA, USA; b Department of Internal Medicine, University of Washington, Seattle, WA, USA; c Clinical Research Division, Fred Hutchinson Cancer Center, Seattle, WA, USA; d Data Core, Shared Resources, Fred Hutchinson Cancer Center, Seattle, WA, USA; e Division of Gastroenterology, Hepatology, and Nutrition, Department of Medicine, University of Minnesota, Minneapolis, MN, USA; f Division of Medical Oncology, Department of Medicine, University of Washington, Seattle, WA, USA; g Translational Science and Therapeutics Division, Fred Hutchinson Cancer Center, Seattle, WA, USA; h Biotechnology Institute, University of Minnesota, St. Paul, MN, USA; i Center for Immunology, University of Minnesota, Minneapolis, MN, USA

**Keywords:** Fecal microbiota transplantation, gut microbiota, hematopoietic cell transplantation, metabolic potential, shotgun metagenomic sequencing

## Abstract

Intestinal dysbiosis has been associated with both the effectiveness and toxicity of immunotherapy in cancer patients, inspiring multiple trials investigating fecal microbiota transplantation (FMT) in these patients. FMT restores microbial community structures damaged by antibiotics and enriches the microbiota with beneficial bacteria. However, the precise mechanism through which FMT exerts its effects and provides clinical benefits remains incompletely understood. Efforts to date have primarily focused on characterizing taxonomic changes following FMT. We hypothesized that FMT may also modify the functional pathways and metabolic capabilities of the gut microbiota, with possible clinical impact. To investigate this, we conducted a study involving 17 patients with blood disorders who received prophylactic FMT from one of the three healthy donors shortly after hematopoietic cell transplantation (HCT). By analyzing shotgun metagenomic profiles of the baseline, pre-FMT, and post-FMT gut microbiota, we demonstrate that FMT effectively restored pathways that had been depleted following HCT. However, it did not significantly reduce pathways that had expanded, indicating that FMT operates primarily through a restorative mechanism, reestablishing lost functional capabilities in the microbiota rather than suppressing overactive pathways. These findings highlight the potential for optimizing FMT protocols and identifying patient populations where FMT may be particularly beneficial.

## Introduction

Fecal microbiota transplantation (FMT) is a holistic therapeutic approach recommended for the treatment of recurrent/refractory *Clostridioides difficile* infection^1^ and with encouraging results in several other dysbiosis-associated conditions.^2–4^ Despite this track record, however, the precise mechanism of action of FMT is unknown. Although donor microbiota engraftment, compositional shifts toward donor, and restoration of a diverse community enriched in beneficial commensal microbiota have been associated with clinical response,^5–8^ there is little evidence to support taxonomic changes *per se* as the main mechanism for clinical benefit. While the new species engrafted from FMT can influence the host via direct contact at the intestinal barrier (e.g., antigenic immune stimulation),^9^ metabolites produced by the new species could also influence the host. A large proportion of fecal and circulating metabolites are indeed produced by the gut microbiota.^10–12^

Conceptually, if a specific metabolic pathway present in a particular species is lost, FMT can restore the pathway by bringing that species back (restorative effect). Alternatively, if a novel pathway emerges during dysbiosis due to the appearance of a new species, FMT can eliminate the pathway through competitive exclusion of that species (decolonization effect). The relative importance of these effects and whether one or both occur with FMT is unknown. The gut microbiota has a high level of functional redundancy owing to its different species carrying overlapping functional repertoires,^13^ an ecological property that increases resilience during perturbation.^14–16^ Functional redundancy allows the niche to lose more members without losing functionality. The functional benefit offered by FMT may depend on functional redundancy of the pre-dysbiosis state and how much of that redundancy has been exhausted during dysbiosis. With a mild loss of function (e.g., due to a non-severe perturbation or high functional redundancy of the original state), the functional restorative effect of FMT might be limited despite microbiota engraftment and taxonomic modifications.

We have used FMT in the prophylactic setting after allogeneic hematopoietic cell transplantation (alloHCT), a curative-intent treatment for several blood disorders. The overarching objective of these studies has been preventing gut microbiota-associated complications, namely infections^17^ and acute graft-versus-host disease (aGVHD).^18^ AlloHCT recipients are heavily exposed to antibiotics in the early post-transplant period,^19,20^ to an extent rarely seen in other clinical settings. They often develop severe dysbiosis,^21^ with a peak around hematopoietic engraftment (2–4 weeks after transplantation)^21^ and associated with increased risk for subsequent aGVHD.^22–26^ We hypothesized that the alloHCT setting provides a unique opportunity to examine the effects of FMT on functional pathways and metabolic potential of the gut microbiota. Our hypothesis is based on two arguments: (i) Severe loss of diversity in the microbiota may exhaust functional redundancy, leading to a significant reduction of functional pathway repertoire. This would potentiate a restorative effect by FMT; (ii) Expansion of pathobionts resulting from compromised colonization resistance, among other mechanisms, would introduce novel pathways, making the microbiota amenable to a decolonization effect by FMT. We administer FMT as soon as possible after hematopoietic engraftment, while dysbiosis is at its peak, increasing the likelihood of detecting an effect in (i) and (ii). Whether and how FMT modulates the metabolic potential of the gut microbiota is clinically relevant because microbiota-derived metabolites can influence aGVHD susceptibility and severity.^27–32^

We initiated a phase II trial in two phases. In the single-arm run-in phase^18^, 3 cohorts of adults undergoing alloHCT each received FMT from one of the 3 healthy stool donors. Treatment was initiated after neutrophil engraftment. The objective was to identify the best donor according to microbiota engraftment and clinical outcomes. In the second phase, patients are randomized between FMT from the best donor from the run-in phase vs. placebo. The primary endpoint is severe aGVHD. Here, we report our findings from preplanned functional analysis of FMT effects on the gut microbiota in the run-in phase. Shotgun metagenomic sequencing allowed us to determine microbiota gene content, from which functional pathways and metabolic potential were inferred. We demonstrate that the primary functional effect of FMT is restorative, with little evidence for a decolonization effect.

## Materials and methods

The trial (ClinicalTrials.gov identifier: NCT06026371) was conducted at Fred Hutchinson Cancer Center (FHCC)/University of Washington (Seattle, WA) and the study protocol was approved by the institutional review board (protocol #RG1123691; FDA IND #29935). Donor screening, stool testing for infectious agents, and product manufacturing (Compound MTP-101-C; FDA IND #15071) were conducted at the University of Minnesota using Good Manufacturing Practices (cGMP) protocols.^33^ Details of donor screening/selection and stool testing,^17^ as well as product manufacturing/storage^34^ have been published. Briefly, compound MTP-101C contains donor microbiota from healthy donors. The microbiota was purified using sequential filtration and washing steps and freeze-dried with 10% trehalose. The stool was donated in a supervised bathroom and transported to the manufacturing facility under the chain-of-custody protocols. The product release criteria included numbers of bacteria and their viability, as determined by LIVE/DEAD BacLight bacterial viability assay (Thermo Fisher Scientific, Waltham MA, USA). The capsules were stored at −80°C until dispensation to the clinical research team. Product stability studies showed no loss of viability at a range of temperatures, including 4°C and room temperature, for at least a month.

Details of the design and conduct of the trial were published recently.^18^ Key elements are summarized in Fig. S1. Briefly, adults undergoing alloHCT using a standard platform in terms of donor type and GVHD prophylaxis were enrolled. No eligibility criteria were used for the underlying disease, conditioning regimen, or graft source. Patients provided written informed consent before conditioning initiation. Following neutrophil engraftment and at least 2 days after discontinuation of antibacterial antibiotics, FMT was initiated. Treatment consisted of three oral capsules of lyophilized microbiota per day for 7 days. Treatment was unsupervised, self-administered at home, with no preparative regimen (e.g. bowel regimen and antibiotic conditioning), and with no changes to concurrent medications. At the time of starting study treatment, all patients were on standard post-transplant immunosuppressive medications as well as prophylactic antiviral and antifungal antibiotics. Three healthy stool donors were used; product from each donor was given to 4–8 consecutive patients (20 total). The microbiota dose per FMT capsule was 1–2 × 10^11^ bacteria, with 91%, 84%, and 81% viability for donors 1, 2, and 3, respectively. Stool samples were collected at baseline (before the initiation of HCT conditioning), pre-FMT (shortly before the first dose of FMT), and post-FMT (4 ± 1 weeks after the last dose of FMT).

Details of microbiota profiling were reported previously.^18^ Briefly, microbiota was profiled by shotgun metagenomic sequencing on an Illumina NovaSeq 6000 using a S2–300 flow cell and a PE150 configuration, with a target sequencing depth of 20 M reads per sample.^18^ Raw reads were quality-processed and host-decontaminated using KneadData v.0.12.0, then inputted to MetaPhlAn4 for species assignment.^35^ Pathways and gene family abundances were profiled using HUMAnN v3.6.^36^ HUMAnN’s tiered search occurs in three phases. First, it identifies community species using MetaPhlAn and its clade-specific marker genes. Then, it maps KneadData-processed reads against the pangenomes of the identified species using Bowtie2.^37^ Finally, it aligns unmapped reads to a comprehensive, non-redundant protein database (EC-filtered UniRef90) using DIAMOND.^38^ HUMAnN performs read-count-based quantification of the microbial gene families and functional pathways present within each sample on both per-species and community-level basis. Pathways were annotated using MetaCyc v24.0 definitions^39^ and gene families using UniRef90 definitions.^40^ HUMAnN’s default reads per kilobase values for gene family and pathway abundances were transformed into copies per million units. For each sample, pathway richness was calculated by counting the number of unique pathways present.

### Statistical analysis

Microbiota profiling and functional analysis were preplanned. Data from all 17 treated patients who provided all three stool samples and were not exposed to antibacterial antibiotics between pre-FMT and post-FMT samples were analyzed. To identify differentially abundant pathways between the three timepoints (baseline vs. pre-FMT vs. post-FMT), pathways were first filtered using relative abundance and prevalence thresholds of 0.01% and 10%, respectively, and then analyzed using general linear model-based Microbiome Multivariable Association with Linear Models (MaAsLin2).^41^ Timepoint was the fixed effect and subject ID was the random effect. Other than a stricter significance threshold of 0.05 than the software’s default 0.25 for corrected *p* values, default parameters were used. The Benjamini–Hochberg method was used to correct the *p* values for multiple testing.^42^

For longitudinal analysis, we applied mixed modeling using the *nlme* package, with subject ID as the random effect and MLE-based inference method to derive *p* values and 95% confidence intervals. In non-longitudinal cases, we used a Wilcoxon’s test to compare continuous variables between two groups and a Kruskal–Wallis test for multiple groups. R v.4.2.0 was used for all analyses.

## Results

**Figure 1.**
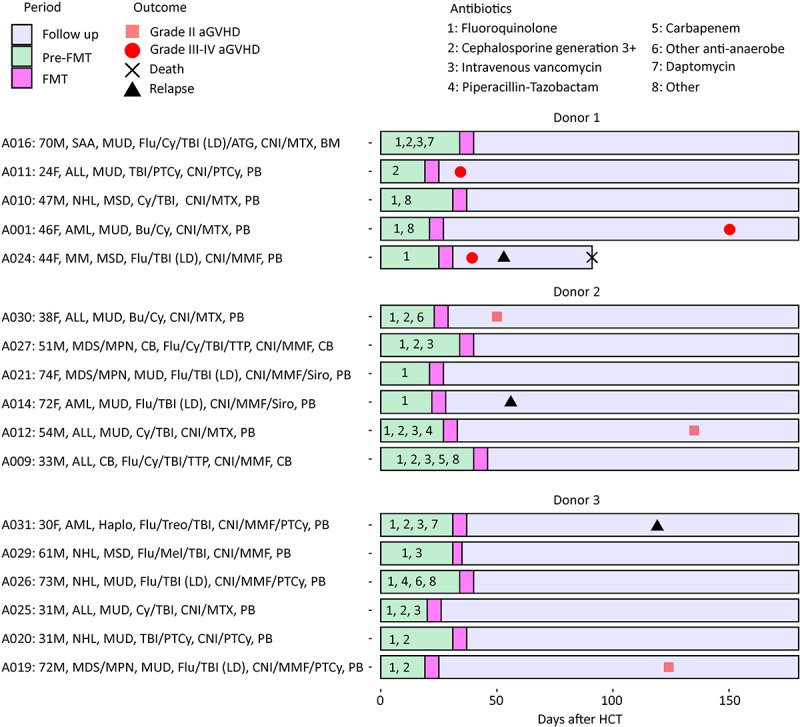
Subject-level patient characteristics and clinical outcomes. Baseline characteristics from left to right include age, sex, disease, donor type, conditioning regimen, GVHD prophylaxis, and graft source. All survivors had 180 days of follow up after HCT. aGVHD: acute graft-versus-host disease; ALL: acute lymphoblastic leukemia; AML: acute myeloid leukemia; BM: bone marrow; CB: cord blood; CNI: calcineurin inhibitor (tacrolimus or cyclosporine); Cy: cyclophosphamide; F: female; Flu: fludarabine; FMT: fecal microbiota transplantation; Haplo: HLA-haploidentical related donor; LD: low-dose; M: male; MM: multiple myeloma; MDS: myelodysplastic syndromes; MMF: mycophenolate mofetil; MPN: myeloproliferative neoplasms; MSD: HLA-matched sibling donor; Mel: melphalan; MSD: HLA-matched sibling donor; MTX: methotrexate; MUD: 9/10 or 10/10 HLA-matched unrelated donor; NHL: non-Hodgkin lymphoma; PB: peripheral blood; PTCy: post-transplantation cyclophosphamide; SAA: severe aplastic anemia; Siro: sirolimus; TBI: total body irradiation; Treo: treosulfan; TTP: thiotepa.

Seventeen patients (10 males; median age 47 years, range 24–74; Figure 1) provided a stool sample at each timepoint (baseline, pre-FMT, and post-FMT), for a total of 51 samples. These patients received FMT from donors 1 (5 patients), 2 (6 patients), and 3 (6 patients). The effects of FMT on microbiota composition and diversity, donor microbiota engraftment, and the association between these and aGVHD were the focus of our previous analysis,^18^ with a summary of key findings provided in Fig. S1. Treatment was safe, with no grade 3+ treatment-related adverse events. Two events meeting dose-limiting criteria but unrelated to study treatment occurred: *Helicobacter pylori* infection (one patient) and *Clostridioides difficile* diarrhea (one patient). Both infections were treated with antibiotics. Further details can be found in our recent publication.^18^ Our focus here is on microbiota functional pathways in 51 patient samples and 3 donor samples. Each donor sample was derived from an actual FMT capsule.

### The primary mode of action of FMT on gut microbiota functional profile is restorative

**Figure 2.**
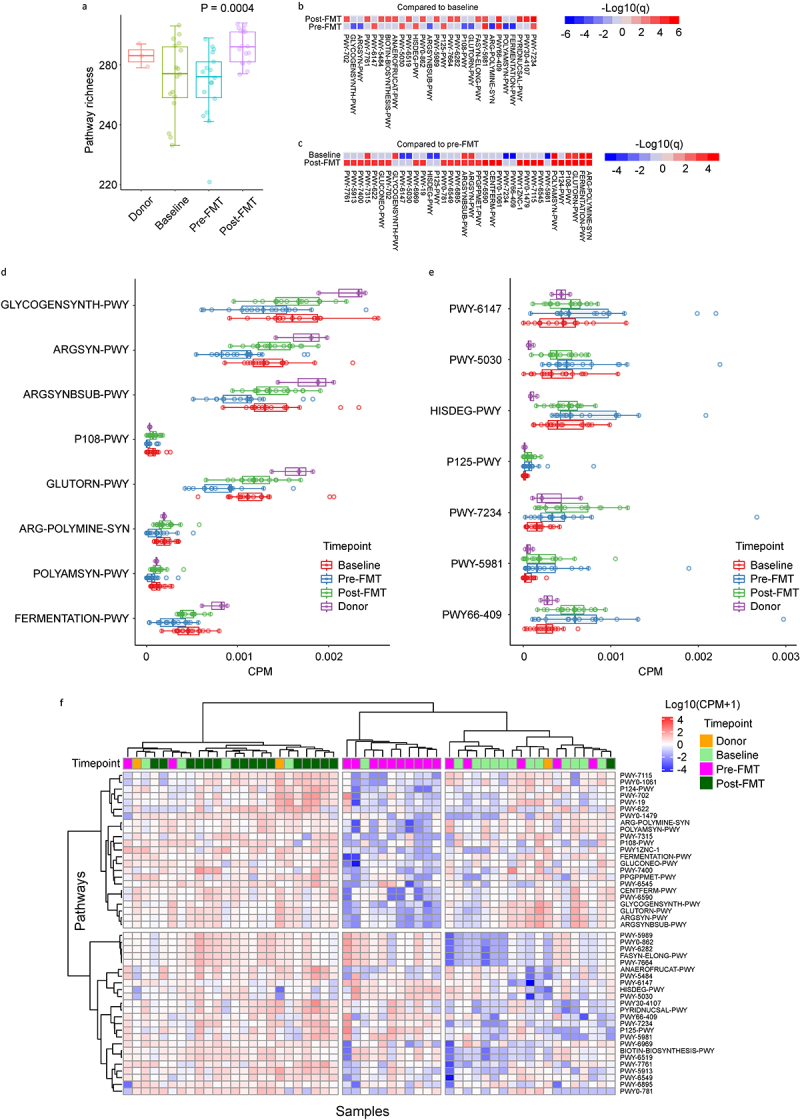
Restorative vs. decolonizing effect of FTM on microbiota function. (a) Pathway richness, defined as the number of unique pathways, in patient samples at different timepoints and donor samples. The *p* value is from a mixed model with timepoint as a fixed effect and subject ID as a random effect. (b-c) the results of MaAsLin2-based differential abundance analysis with subject ID considered a random effect. Panel (b) compares pre-FMT and post-FMT samples to baseline, while panel (c) compares post-FMT and baseline samples to pre-FMT. Red and blue cells indicate significantly expanded and depleted pathways, respectively. Color intensity shows the level of statistical significance. A Benjamini-Hochberg q value < 0.05 was used to define statistical significance. (d) Comparison between patient samples at different timepoints and donor samples for pathways that were significantly depleted in pre-FMT samples compared to baseline (blue cells in the second row of panel b). q values corresponding to the change from pre-FMT to post-FMT are per panel (b) and all but one (GLYCOGENSYNTH-PWY; q = 0.054) reached the significance threshold. (e) Comparison between patient samples at different timepoints and donor samples for pathways that were significantly enriched in pre-FMT samples compared to baseline (red cells in the second row of panel b). q values corresponding to the change from pre-FMT to post-FMT are per panel (b) and none reached the significance threshold. (f) Pathway heatmap visualizing the results of unsupervised hierarchical clustering using log10-transformed, CPM-normalized abundances (blue-red gradient) and a ward.D function. Pathways used to generate the plot are those in panels (b) and (c) that reached statistical significance. Each column is a sample, and each row is a pathway.

A total of 277 pathways were identified in patient samples (Table S1). Although pathway richness (i.e. the number of unique pathways present in a sample) did not change from baseline to pre-FMT, it increased after FMT (p = 0.0004), reaching donor levels (Figure 2(a)). In MaAsLin2-based differential abundance analysis and with subject number considered a random effect, pre-FMT samples were significantly enriched in seven pathways and depleted in eight pathways when compared to baseline (Figure 2(b)). Post-FMT samples were significantly enriched in 16 pathways compared to baseline (Figure 2(b)) and 28 pathways compared to pre-FMT (Figure 2(c)). No pathway was enriched in baseline or pre-FMT samples compared to post-FMT. The heatmaps in Figures 2(b,c) indicate post-FMT expansion of seven of the eight pathways depleted in pre-FMT samples compared to baseline. The remaining pathway (GLYCOGENSYNTH-PWY; glycogen biosynthesis from ADP-D-Glucose) also expanded after FMT (p = 0.004), but due to its corrected *p* value of 0.054, it did not appear as a significantly expanded pathway on the heatmap. In contrast, none of the seven pathways that had significantly expanded in pre-FMT samples compared to baseline declined after FMT. These findings, summarized in Figures 2(d,e) show that the primary effect of FMT on gut microbiota function was restorative rather than decolonizing. Four of the eight restored pathways were involved in arginine/polyamine biosynthesis (see below). Expanded pathways before FMT which were not depleted following FMT included purine salvage and histidine degradation pathways known to be upregulated under nutrient-poor conditions (e.g. those expected to occur in the colonic lumen early after HCT). In hierarchical clustering analysis using significant pathways in differential abundance analysis (Figure 2(f)), samples formed three distinct clusters, representing different stages of injury and recovery. From left to right, the three clusters were enriched in post-FMT, pre-FMT, and baseline samples, respectively.

### Donor pathways robustly engraft in patient microbiota after FMT

**Figure 3.**
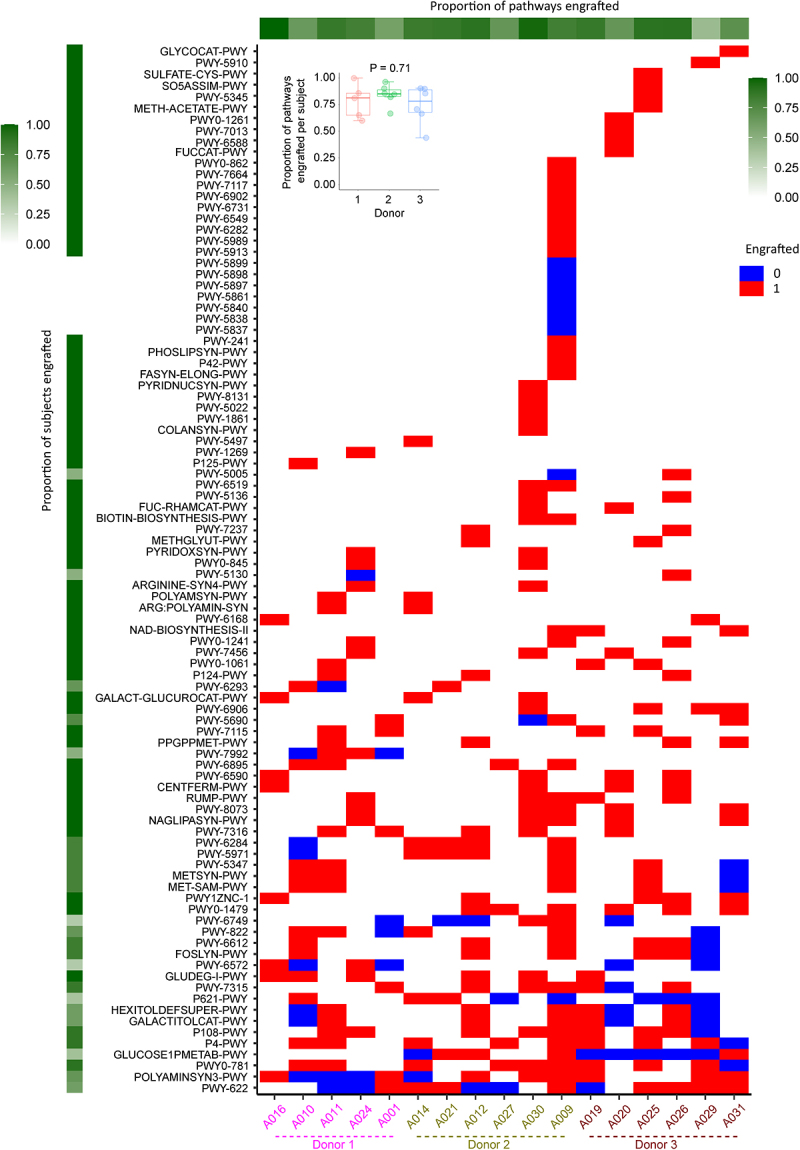
Pathway engraftment analysis. Engraftment of each donor pathway (rows) in each patient (columns) is shown. Patients are sorted according to their donors. For each donor and pre-FMT sample pair, with the pre-FMT sample being from a patient treated with FMT from the donor in the pair, we considered a pathway engraftable if it was present in the donor and absent in the pre-FMT sample. Red and blue cells indicate engraftable pathways that engrafted and did not engraft, respectively. Non-engraftable pathways are shown as white cells. Pathways are sorted according to the number of donor/pre-FMT sample pairs in which they were considered engraftable, with those in the bottom of the plot being more frequently engraftable. For each pathway considered engraftable in at least one pair, the proportion of subjects in whom the pathway engrafted was determined and defined as per-pathway engraftment rate. Per-pathway engraftment rates are shown along the column sidebar on the left. For each patient, the proportion of engraftable pathways that appeared in the post-FMT sample (i.e. engrafted) was determined and defined as per-patient pathway engraftment rate. Per-patient pathway engraftment rates are shown along the row sidebar at the top. The inset shows the proportion of engraftable pathways that engrafted in patients treated with each donor. The *p* value is from a Kruskal–Wallis test. Each box shows the median (horizontal middle line) and interquartile range. Whisker lines indicate non-outlier maximum and minimum values. A small jitter is included for better visualization.

Next, we examined the engraftment of donor pathways using a simple metric. For each donor and pre-FMT sample pair, with the pre-FMT sample being from a patient treated with FMT from the donor in that pair, we defined engraftable pathways as those present in the donor that were absent in the pre-FMT sample. For each patient, the proportion of such pathways that engrafted (i.e. appeared in the post-FMT sample) was determined and defined as per-patient pathway engraftment rate. For each pathway considered engraftable in at least one sample pair, the proportion of subjects in whom the pathway engrafted was determined and defined as per-pathway engraftment rate. Figure 3 shows the results of this analysis. Of the nine pathways that were engraftable in >50% of patients (pathways in the lower parts of Figure 3), the three top engrafting were the aspartate superfamily (PWY0 − 781; engrafted in 9/10 patients), pyruvate fermentation to propanoate I (P108-PWY; engrafted in eight out of patients), and superpathway of L-lysine, L-threonine, and L-methionine biosynthesis I (P4−PWY; engrafted in eight out of patients). In contrast, glucose and glucose-1-phosphate degradation (GLUCOSE1PMETAB-PWY; engrafted in 4/10 patients) and nylon-6 oligomer degradation (P621-PWY; engrafted in three out of eight patients) engrafted relatively poorly. The median pathway engraftment rate across all patients was 0.85 (range 0.44–1.00), with no difference according to the specific donor used (*p* = 0.71; Figure 3 inset).

### Emergence of unique donor pathways requires FMT

Next, we asked whether FMT was necessary for the emergence of unique donor pathways (i.e. those present in the donor but not in the pre-FMT sample), while also entertaining an alternative scenario where other sources including diet were predominantly responsible for this recovery. To investigate this question, we selected donor pathways that were absent in at least 1 pre-FMT sample, resulting in 94 pathways and 349 triads composed of a donor sample, a pre-FMT sample, and a pathway. In each triad, the pre-FMT sample belonged to the patient treated with the donor in that triad, and the pathway was either present (305 triads) or absent (44 triads) in the donor but was absent in the pre-FMT sample. The pathway absent in the pre-FMT sample recovered in the post-FMT sample in 245 of the 305 instances where the pathway was present in the donor. This was significantly higher than the observed recovery in only 25 of the 44 instances where the pathway was absent in the donor (recovery rate 80% vs. 57%, respectively; *p* = 0.001 from a chi-square test).

### FMT-dependent recovery of metabolic pathways is predominantly due to some of the less dominant species contributors in the FMT product

**Figure 4.**
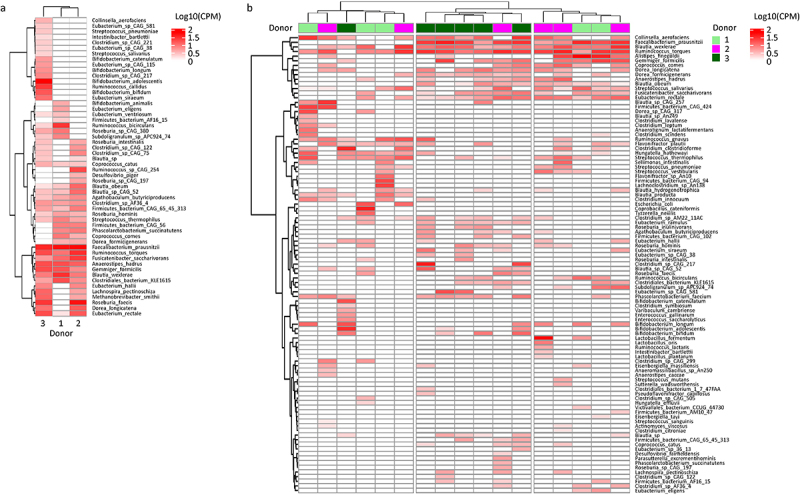
Species-level contribution in donor and post-FMT samples to the pathway of L-arginine biosynthesis via L-ornithine. A case study was performed for ARGSYN-PWY due to its clinical relevance and large number of contributing species. Each column is a sample, and each row is a species. Each cell is colored on a white to red gradient according to the log10-transformed copies per million (CPM) of the entire pathway in the corresponding sample that is assigned to the corresponding species. Heatmaps visualize the results of unsupervised hierarchical clustering using a ward.D function. (a) Heatmap of the three donor samples, with donor 3 clustering separately from donors 1 and 2. (b) Heatmap of post-FMT patient samples. Donors used for the corresponding patients are shown at the top. Post-FMT samples in patients treated with donor 3 form a distinct cluster, separate from donors 1 and 2.

As a case study, we then determined species-level contribution in donor and post-FMT samples to a significantly expanded pathway after FMT that is thought to be of clinical relevance. We focused on the ARGSYN-PWY pathway (L-arginine biosynthesis via L-ornithine) because of its presence in many bacterial species and connection with polyamine biosynthesis through which L-arginine is converted to putrescine and spermidine. Polyamines have critical roles in gut epithelial renewal and healing after damage as well as gut barrier integrity and function.^43^ ARGSYN-PWY was severely depleted before FMT but recovered after FMT. HUMAnN’s pathway abundance tables (normalized to copies per million) include the number of copies of the entire pathway of interest assigned to each species which we used to examine whether FMT influences the spectrum of species-level contributions to pathways. We argued that if this hypothesis were true, we would see donor-specific clustering of post-FMT samples based on species contributions to the given pathway. The three donors had different spectra of species distributions to ARGSYN-PWY, with donor 3 clustering separately from donors 1 and 2 (top dendogram in Figure 4(a)). The top three contributing species in donor 1 were *Faecalibacterium prausnitzii*, *Blautia wexlerae*, and *Ruminococcus bicirculans*. In donor 2, these species were *Faecalibacterium prausnitzii*, *Roseburia faecis*, and *Ruminococcus torques*. Finally, the top three contributing species in donor 3 were *Bifidobacterium adolescentis*, *Faecalibacterium prausnitzii*, and *Roseburia faecis*. Several *Bifidobacterium* species were among top contributors to ARGSYN-PWY in donor 3 but made no contribution in donors 1 or 2. Comparing the three donors for their genus- and species-level microbiota composition, we previously reported that *Bifidobacterium* and its several species (esp. *B. bifidum* and *B. adolescentis*) were enriched in donor 3.^18^ In hierarchical clustering analysis using species contributions to ARGSYN-PWY, post-FMT samples from patients treated with donor 3 clustered together (middle cluster in Figure 4(b)). Interestingly, donor 3 was the donor with the greatest species engraftment rate and best clinical outcomes in her corresponding patients.^18^

Some top contributing species in donors (e.g. *Faecalibacterium prausnitzii*, *Blautia wexlerae*, and *Ruminococcus torques*) were also top contributors to ARGSYN-PWY in post-FMT samples regardless of the specific donor used and thus did not contribute significantly to the observed donor-specific clustering. Our findings from engraftment analysis in the previous report^18^ suggested that natural recovery of the microbiota upon declining antibiotic pressure and normalization of diet, rather than FMT, was the main underlying mechanism for the recovery of these species. For example, 70% of *Blautia wexlerae* and 65% of *Faecalibacterium prausnitzii* in post-FMT samples were new strains that were not present in pre-FMT or donor samples and likely originated from diet. Similarly, 71% of post-FMT *Ruminococcus torques* were patient-specific strains (i.e. recovery of baseline microbiota), thus their contribution to ARGSYN-PWY was not FMT-dependent. Together, these findings suggest that FMT-dependent recovery of depleted metabolic pathways is predominantly due to some less dominant species contributors in the FMT product which are likely more donor-specific. The more robust microbiota engraftment from donor 3 compared to donors 1 and 2 likely led to donor three-specific pathway-based clustering of post-FMT samples.

### Post-FMT microbiota functional pathways were not associated with future aGVHD

Finally, we examined in exploratory analysis whether gut microbiota functional pathways in post-FMT samples were associated with subsequent development of severe aGVHD. In this MaAsLin2-based differential abundance analysis, we found no significantly depleted or enriched pathways in the 3 patients who later developed severe aGVHD vs. the 14 who did not.

## Discussion

In this preplanned analysis of the run-in phase of a randomized placebo-controlled trial in alloHCT recipients, we demonstrate that the main mechanism of action of FMT on gut microbiota metabolic function is restorative rather than decolonizing. FMT was highly effective in restoring pathways depleted as a result of early post-transplant exposures (e.g., antibiotics). The overall extent of pathway recovery did not depend on the specific donor used. Several pathways that were markedly depleted after HCT but effectively restored following FMT were involved in arginine and polyamine biosynthesis which enhance gut barrier integrity, function, and repair after damage. Polyamine biosynthesis was one of the five pathways significantly enriched after FMT in a previous trial in adolescents with obesity.^44^ Cytotoxic damage to the intestinal epithelial barrier is an early trigger for subsequent aGVHD.^45–47^ Restoration of polyamine production by the gut microbiota might be a mechanistic pathway by which FMT can help prevent aGVHD. As opposed to its strong restorative efficacy, FMT appeared unable to reduce the pathways expanded between baseline and pre-FMT timepoints. These included pathways used by bacteria (esp. pathogenic species) to survive nutrient-deplete conditions. This observation may be explained by the indispensable fitness gain provided by the enriched pathways to the species harboring them in the harsh colonic luminal environment characterized by altered oxygen tension, antibiotic presence, rapid transit time due to commonly present diarrhea, insufficient presence of dietary material, and ongoing mucosal injury. A more favorable ecosystem in the setting of obesity in the previous FMT trial probably rendered the enriched pathways less critical for fitness, leading to the observed declines after FMT.^44^

We evaluated whether and how donor microbiota engraftment influences functional properties of the gut microbiota. In a case study focusing on the pathway of L-arginine biosynthesis via L-ornithine as a potentially relevant pathway for GVHD pathogenesis, post-FMT species contributions to this pathway in patients treated with product from donor 3 resembled species contributions in donor 3. Intriguingly, this resemblance was apparent only for donor 3, the donor with the greatest microbiota engraftment and yielding the best clinical outcomes.^18^ In addition, the results of strain-level engraftment analysis in our previous work combined with the functional results here showed that the top contributing species in post-FMT samples were predominantly of a non-donor origin, whereas several of the less dominant contributors were donor-derived. This finding suggests that while the recovery of a pathway *per se* and its main contributing species may not depend on the donor, its less dominantly contributing species are determined by the donor, particularly when microbiota engraftment is sufficiently strong. In the only other study evaluating the relationship between donor microbiota engraftment and gut microbiota metabolic function,^44^ engrafted strains altered not only the taxonomic composition of the microbiota, but also its metabolic potential. The results of this trial in adolescents with obesity suggest that functional alterations induced by FMT are specific to the genes present in engrafting strains, especially those derived from the dominant donors. Our findings are consistent with these results.

With a few exceptions, human FMT studies thus far have focused on taxonomic changes and taxonomy-derived indices such as diversity and engraftment to describe the effects of FMT on the gut microbiota. In patients with *C. difficile* infection, FMT leads to restoration of gut microbiota-mediated bile metabolism and short-chain fatty acid production, both linked to clinical efficacy of FMT.^48–51^ Improved bile acid deconjugation and secondary bile acid formation by the gut microbiota have also been observed after FMT in patients with cirrhosis.^52^ In another study, several amino acid biosynthesis pathways were expanded following FMT in patients with irritable bowel syndrome (IBS).^53^ Finally, in a study in adolescents with obesity, FMT led to enrichment of nicotinamide adenine dinucleotide metabolism, polyamine production, vitamin synthesis (menaquinones and tetrapyrroles), and L-lysine metabolism, with concomitant depletion of pantothenate, coenzyme A, and peptidoglycan biosynthesis pathways.^44^ We provide further evidence, the first from an early post-alloHCT setting, for FMT-induced alterations of gut microbiota metabolic potential. The extent to which these alterations are important for clinical effects of FMT is unknown and requires further research.

FMT effects on metabolic function of the microbiota may be modified by interventions targeting the patient and/or donor. As an example, a brief course of non-absorbable antibiotics such as oral vancomycin before FMT has been shown to improve microbiota engraftment.^6,54^ This approach might also enhance the introduction of new pathways and/or reduction of expanded pathways, concepts to be explored in future work. In addition, competition between the incoming donor’s taxa and the patient’s taxa for the same nutrients could lead to decolonization.^55–59^ While the selection of donors in this trial was based on FDA-mandated safety criteria and not microbiome-based, future efforts could incorporate additional criteria including the presence of taxa competing with patient’s taxa in nutrient access and utilization. Such efforts would help make FMT a precision therapeutic.

Both diet- and microbiota-derived metabolites in the gut have been implicated in aGVHD pathogenesis. Pertinent microbial metabolites include butyrate,^27^ bile acids,^30–32^ tryptophan metabolites,^31^ and phenyllactic acid.^29^ Pertinent dietary metabolites include xylose^60^ and lactose.^25^ Overall, we did not find strong evidence for the involvement of FMT-induced microbiota functional changes in aGVHD pathogenesis, though this analysis was limited by the small number of events (three patients with the clinical outcome). The importance (or lack thereof) of functional restoration for a clinical benefit seems to be context dependent. In a previous trial of FMT in patients with IBS, improvements in microbiota functional pathways did not correlate with symptom relief.^53^ In contrast, restoration of gut microbiota-mediated vitamin B6 metabolism after human- derived FMT alleviated social deficits in a mouse model of autism.^61^ Larger studies are needed to determine whether restoration of microbial metabolites following FMT has a protective effect against aGVHD.

This work has some limitations. First, the sample size was modest. Therefore, although the findings are novel and thought-provoking, they need to be validated in other cohorts. Second, the inherent heterogeneity of clinical status in patients undergoing alloHCT might have influenced the results by affecting the efficacy of FMT on microbiota composition, metabolic pathways, and engraftment. A randomized trial, such as the ongoing second phase of this trial, would minimize these effects and increase the signal-to-noise ratio.

In conclusion, we administered FMT in a prophylactic setting early after alloHCT, a clinical scenario in which major disruptions to gut microbiota-host hemostasis commonly occur. Our previous analyses and those presented here demonstrate efficacy of FMT in restoring the commensal gut microbiota, reducing pathobionts, and restoring the microbiota functional repertoire as represented by its gene content and metabolic potential. In contrast, FMT had little effect on pathways that had expanded between baseline and pre-FMT timepoints. Our ongoing randomized placebo-controlled trial using the best donor from the run-in phase as the sole stool donor will determine whether taxonomic and functional effects of FMT lead to a clinical benefit. Our results have implications beyond the transplantation field. As an example, when the dysbiotic state is characterized primarily by a loss of beneficial pathways and metabolites, FMT could be an effective treatment. In contrast, when eliminating expanded pathways and metabolites from the gut microbiota is the main objective, especially in the presence of severe pathology where the microbial fitness advantage conferred by the expanded pathways is likely substantial, FMT alone may not be as effective. Such patients may be best treated with other or combination microbiota therapeutics.

## Supplementary Material

Supplemental Material

Figure_S1.tiff

## Data Availability

The fastq sequencing files are available from the NCBI Sequence Read Archive, BioProject ID PRJNA1209112. Functional pathways and their community- and species-level abundances in each sample are provided in Table S1. De-identified subject-level clinical metadata are available in Figure 1 and can be linked to the sequencing and pathway files. This paper does not report original code. Any additional information required to reanalyze the data reported in this paper is available from the corresponding author upon request.
