# Supplementary figures and images for: Effect of fecal microbiota transplantation on gut microbiota functional profile in recipients of allogeneic hematopoietic cell transplantation
Source: Gut Microbes. 2025 Aug 27;17(1):2551882. doi: 10.1080/19490976.2025.2551882 (PMC12396129; doi:10.1080/19490976.2025.2551882)

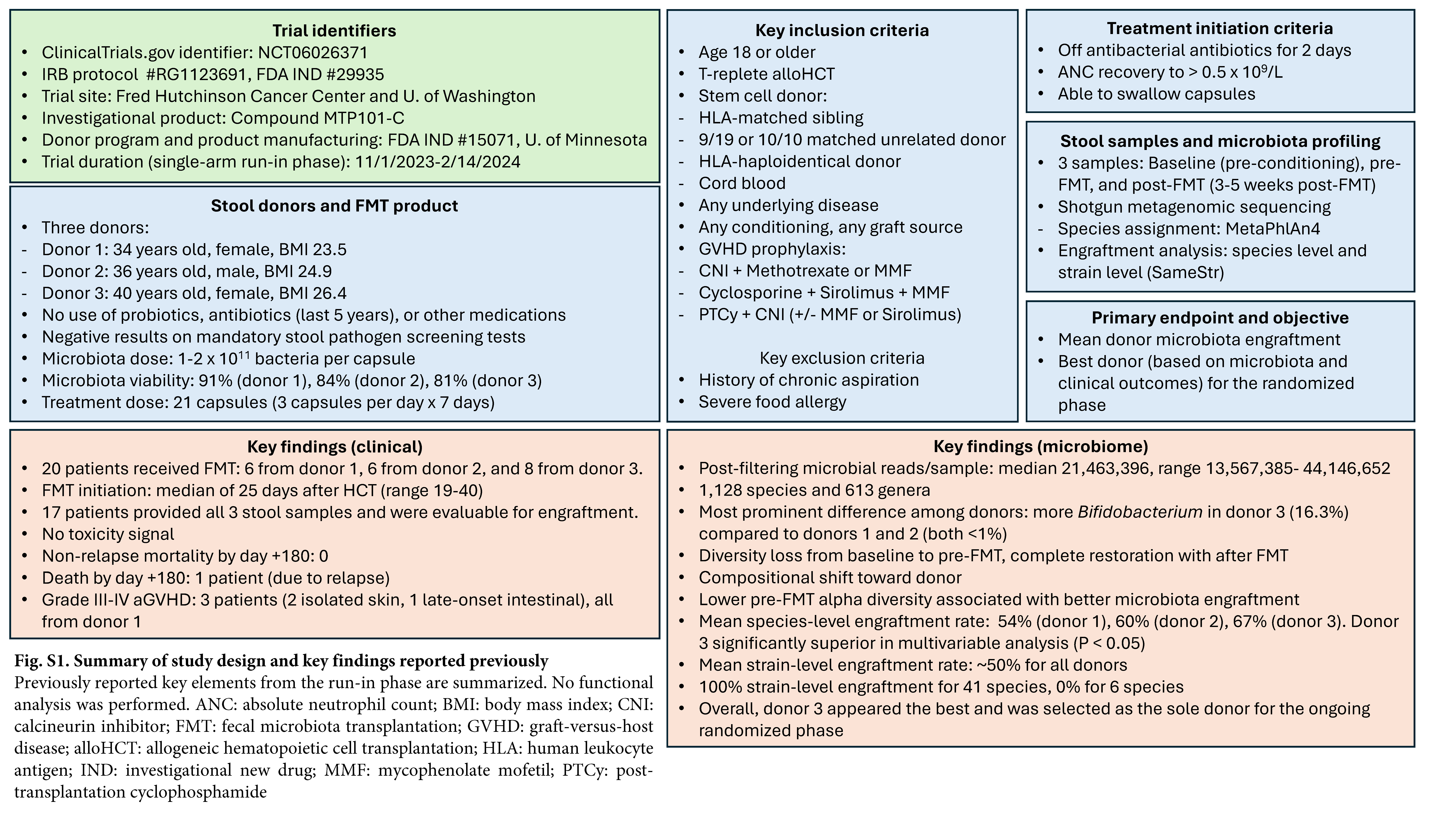

Supplement: Figure_S1.tiff [file KGMI_A_2551882_SM8140.tiff]
